# Necrotizing sialometaplasia on the hard palate after sequential induced vomiting: case study

**DOI:** 10.1016/j.bjorl.2023.101385

**Published:** 2023-12-28

**Authors:** Elaine Costa, Gabriel Caetano de Jesus, Bruno Siqueira Bellini, Ana Cristina Coelho Dal Rio Teixeira

**Affiliations:** a Departamento de Otorrinolaringologia e Cirurgia de Cabeça e Pescoço, Universidade Estadual de Campinas (UNICAMP), Campinas, SP, Brazil; b Odontologia, Hospital de Clínicas, Universidade Estadual de Campinas (UNICAMP), Campinas, SP, Brazil

## Introduction

Necrotizing sialometaplasia (NS) is a rare, benign, and self-limiting condition with unclear incidence and prevalence rates, though many studies report an incidence below 1%. Shin et al. found a 0.06% incidence of NS among all oral biopsies.^1^ It occurs more commonly in men, with a male-to-female ratio of 1.95:1. The age range is vast, from 1.5 to 83 years old, though most cases present above the fourth decade.^2^ However, patients with bulimia who develop NS are often younger women between 20 and 40 years old.^3^

The misdiagnosis of this condition may occur, as NS can mimic malignant tumors like mucoepidermoid and squamous cell carcinoma on clinical and histological examination.^1^, ^2^ Therefore, appropriate diagnosis is essential for managing and avoiding unnecessary invasive procedures. Careful clinicopathological correlation and immunohistochemical analysis can help differentiate NS from concerning entities.

This case report describes a spontaneously appearing ulcerated lesion on the posterior hard palate. During the diagnostic investigation, we observed an association with local trauma following oral cavity manipulation to induce vomiting. The histopathology confirmed the diagnosis of necrotizing sialometaplasia. Through supportive clinical treatment, we observed complete healing in the 2-month follow-up.

## Case report

**Figure 1:**
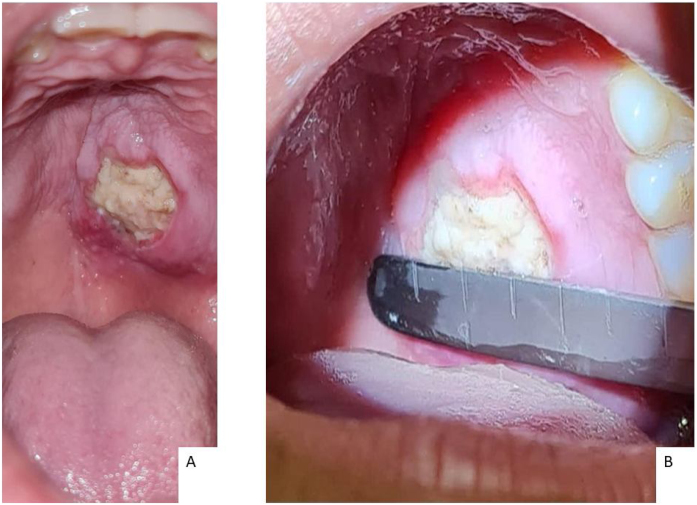
Ulceration at initial assessment (10th day of onset of symptoms). (A) Ulceration on the hard palate posteriorly, on the left, and near the midline, with central necrotic tissue and perilesional edema without erythema or bone exposure. (B) Dimensions 2.0 × 2.5 cm.

A female, 21 years old, previously healthy, sought outpatient care with an otorhinolaryngologist due to a lesion on the hard palate from onset ten days ago. Its initial presentation was a macula associated with local paresthesia, progressively evolving to ulceration, with self-limited bleeding and halitosis. Physical examination identified a 2.0 × 2.5 cm lesion on the left side of the hard palate with central necrotic tissue and perilesional edema. It did not cross the midline and spared the gums. No signs of bone exposure or oronasal fistula were present (Fig. 1).

She took non-steroidal anti-inflammatory drugs for pain relief and used mouthwash with 0.12% Chlorhexidine. She reported self-induced daily emetic episodes associated with a depressive condition weeks before the appearance of the lesion. There was no history of psychoactive substance abuse, oral sex activity, contact with venomous animals, associated skin lesions, the appearance of genital lesions, discharge, or dysuria. The patient owned a domestic cat with up-to-date vaccinations.

**Figure 2:**
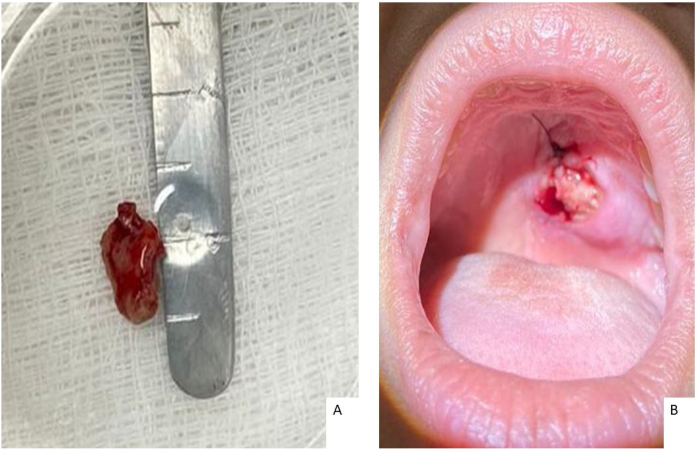
Biopsy of the lesion. (A) 1.5 × 1.0 cm fragment. (B) Biopsy area sutured, fragment obtained from the edges in a transition area with sick and normal tissue and an adequate depth.

The patient underwent a biopsy with local anesthesia during the first consultation for histopathological and culture analysis (Fig. 2). The team recommended support measures and provided guidance and referral for evaluation with the mental health unit without further induced vomiting during follow-up.

**Figure 3:**
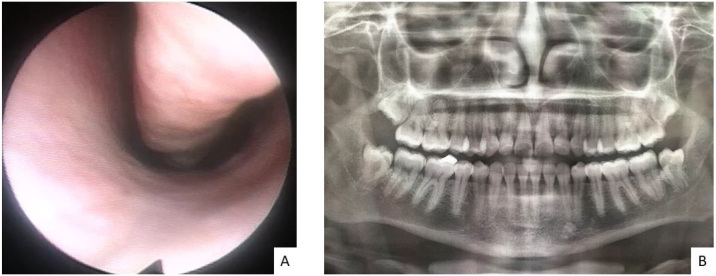
Complementary exams. (A) Flexible nasolaryngoscopy shows the inferior meatus of the left nostril. No signs of fistula on the nasal floor. (B) Panoramic radiograph of the face without bone erosion.

**Figure 4:**
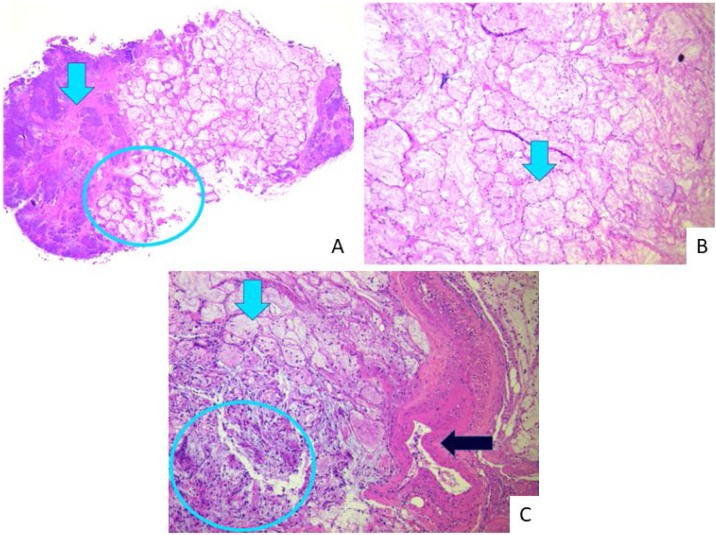
Histopathology. Extensive infarction of mucous salivary glands and adjacent tissues with several thrombosed vessels in different stages of organization. (A) Arrow indicates coagulative necrosis; circle: interface with acinar necrosis. (B) Arrow shows necrosis with preserved acinar architecture. (C) Blue light arrow: acinar necrosis; circle: islands of squamous metaplasia; black arrow: vascular thrombosis.

**Figure 5:**
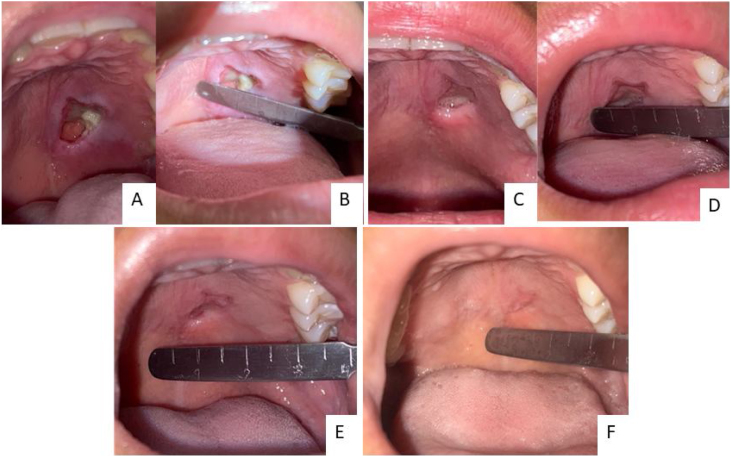
The evolution of the lesion shows progressive regression until complete healing. (A and B) 17th day since the onset of symptoms. (C and D) 24th day. (E) 38th day. (F) 63rd day.

Assessment of both nostrils' inferior meatus through nasolaryngoscopy did not evidence a fistula in the nasal floor. Panoramic radiograph of the face without signs of bone erosion (Fig. 3). The result of cultures for fungi and bacteria was negative, and histopathology showed extensive infarction of the mucous salivary glands and adjacent tissues with several thrombosed vessels in different stages of organization (Fig. 4). The patient presented progressive lesion reduction until complete healing in the 2-month follow-up (Fig. 5).

## Discussion

Necrotizing sialometaplasia is associated with several risk factors, including local trauma, surgery, radiation, inflammation, and bulimia. The condition involves necrosis of salivary gland tissue, leading to ulceration. The most widely accepted theory to explain the pathogenesis of NS is that it represents an ischemic event affecting the vasculature supplying the salivary glands, triggered by local mechanical, chemical, or thermal injury.^2^, ^3^

It may present with pain, paresthesia, and circumscribed ulceration 1–3 cm in diameter with edematous and erythematous edges.^2^, ^3^ It usually affects the posterior hard palate, but other areas that contain minor or major salivary glands in the oral cavity can be affected. Eventually, it can present as intact mucosa with a floating region.^2^ This self-limiting lesion resolves in 2 or 3 months with supportive measures. Usually, it has a good prognosis, without recurrences after healing.^3^

Diagnosis requires detailed anamnesis and adequate biopsy sampling. Histopathologically, it evidences coagulative necrosis with preserved architecture, squamous metaplasia, and eventually superimposed dysplasia. It makes a differential diagnosis with squamous cell, mucoepidermoid, and cystic squamous cell carcinoma.^1^ There are reports of misdiagnosis as malignancy in 21% of preoperative biopsied cases, which could lead to unnecessary invasive procedures in patients where NS is the correct diagnostic.^2^

It can be challenging to determine the relationship between self-induced vomiting and oral ulcers in patients without a formal bulimia diagnosis. A careful and empathetic approach during the clinical history may help these patients feel comfortable reporting self-induced vomiting behaviors. There appears to be a plausible causal link between chronic vomiting and the development of NS. The chemical irritation from repeated exposure to stomach acid during self-induced vomiting can lead to erythema and oral mucosal irritation, mainly affecting the pharynx and palate. Additionally, mechanical trauma from repetitive insertion of fingers or objects to trigger the gag reflex and vomiting may contribute. These chemical and mechanical insults likely damage the vasculature supplying the salivary glands, resulting in ischemic necrosis or infarction of the glandular tissue.^4^, ^5^

In the case discussed here, the representative biopsied area analyzed by an experienced pathology team was elucidative. Thus, understanding clinicopathologic findings is essential to avoid unnecessary intervention during management. Also, once we identified risk factors, such as self-induced vomiting, we offered support and specific orientation to prevent future occurrences. Schöning et al. described two cases of necrotizing sialometaplasia on the hard palate after chronically self-induced vomiting.^4^

## Conclusion

Healthcare professionals must thoroughly evaluate oral ulcerations through detailed anamnesis, risk factor analysis, and biopsy. A careful histopathological examination is critical for accurate diagnosis and avoiding misdiagnosis of oral ulcerative lesions, especially when suspected of necrotizing sialometaplasia. The variety of potential differential diagnoses necessitates this diligent approach. Furthermore, patient guidance during follow-up visits is essential to facilitate proper management and modification of risk factors, preventing unnecessary invasive interventions.

## Conflicts of interest

The authors declare no conflicts of interest.
